# Testing a Potential Averaging for Quantum Inelastic Dynamics

**DOI:** 10.1021/acs.jpca.6c03968

**Published:** 2026-09-23

**Authors:** K. Giri, S. Rana, U. Lourderaj, N. Sathyamurthy, E. Yurtsever, K. Dulitz, F. A. Gianturco

**Affiliations:** † Department of Computational Sciences, 231505 Central University of Punjab, Bathinda, Punjab 151401, India; ‡ School of Chemical Sciences, 193155 National Institute of Science Education and Research (NISER) Bhubaneswar, Khurda, Odisha 752050, India; § 124268 Indian Institute of Science Education and Research Mohali, SAS Nagar, Punjab 140306, India; ∥ Department of Chemistry, 52979 Koc University, 34450 Istanbul, Turkey; ⊥ Insitut für Ionenphysik und Angewandte Physik, 27255 Universität Innsbruck, 6020 Innsbruck, Austria; # Institut für Ionenphysik und Angewandte Physik, Universität Innsbruck, 6020 Innsbruck, Austria; ∇ Homi Bhabha National Institute (HBNI), Training School Complex, Anushakti Nagar, Mumbai 400094, India

## Abstract

To obtain the inelastic cross sections for rotation state-changing processes, two crucial ingredients are required: the intermolecular forces from *ab initio* quantum chemical methods and the full coupled channels (CC) treatment of the quantum scattering. The present work supports earlier work that there is a possible path which reduces the computational demands by simplifying the multidimensional potential energy surface while using the exact CC quantum dynamics to extract scattering attributes. This is applied successfully to the case of ^11^BN^–^(*X* ^2^Σ^+^, *j*_1_) in collision with H_2_(*X* ^1^Σ_*g*_^+^, *j*_2_ = 0), the former being an anion of interest in laser cooling at cryogenic temperatures. The exact CC calculations of the quantum dynamics, using either the full four-dimensional (4D) potential energy surface (PES) or its two-dimensional (2D) averaging (discussed in the main text), show that quantum attributes from the two procedures turn out to be essentially the same. These findings indicate that the computational savings attained by reducing the dimensions of the PES while keeping the quantum dynamics exact are a realistic option for the case of the hydrogen molecule in its rovibrational ground state as a partner in low-temperature collision processes with molecular ionic species.

## Graphical abstract

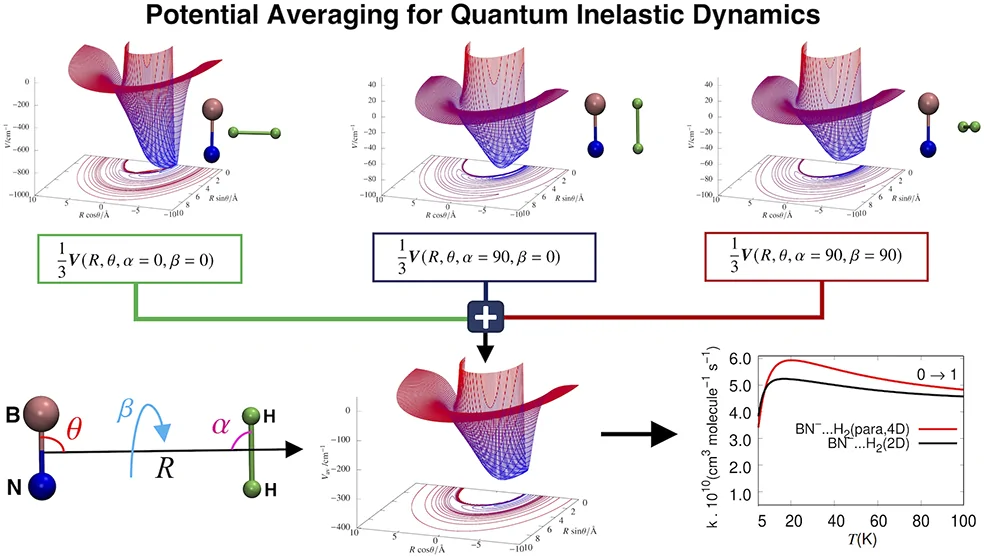

## Introduction

One of the computationally demanding tasks when studying collision-driven internal energy transfers in molecules and ions is to accurately describe the structural forces at play and then perform the exact evaluations of the corresponding state-to-state quantum cross sections over the relevant range of collision energies. Such quantities are, in fact, essential for the determination of the individual inelastic rate coefficients, describing the collision efficiency of the processes and linked to modeling the evolutionary kinetics of the relative populations of those internal states.

Under the realm of low-temperature phenomena, either in cold traps or under interstellar medium (ISM) conditions, one is generally considering cooling kinetics up to about 100 K and chiefly state-changing collisions involving rotational state populations. Furthermore, the most likely partners for the collisional cooling of trapped ions would be the standard buffer gases employed there, i.e., He or H_2_ in its usual mixture of *ortho*- and *para*-H_2_. These two partners also happen to be the most abundant baryons of interest in molecular clouds, in dark or diffuse clouds, and therefore also the most likely to be employed when modeling internal rotational state changing in the neutral or ionic species detected in that astrochemical environment.^1,2^

From the computational viewpoint, one therefore needs to obtain as accurately as possible: (i) the multidimensional description of the quantum forces from quantum chemistry codes, and (ii) the quantum dynamical treatment of the inelastic couplings which generate the needed large variety of cross sections and rate coefficients for the kinetic modeling of the population evolution of such internal states, either in cryogenic ion traps or in the ISM. These tasks are both computationally very demanding and therefore, over the years, various attempts have been made in reducing the required efforts either by simplifying the description of the potential energy surface (PES) guiding the dynamics or reducing the number of coupled channels in the case of time-independent quantum dynamics.^3,4^ In the present study, we show further evidence that, at least in the case of the most common molecular partner in both environments, i.e., the H_2_ molecule in its ground rotational state, it is possible to substantially reduce the computational effort without either simplifying the quantum dynamics, or using a smaller basis set expansion for the PES evaluation. We shall suggest below to perform instead a physics-driven dimensionality-reduction of the relevant PES obtained from accurate quantum chemistry methods. This is a different path than the one often suggested of reducing the CC equations.^3,5^

Recent advances from the new generation of telescopes are, in fact, providing an unexpectedly large volume of observational data, directed at astrophysical environments ranging from the interstellar medium to the atmospheres of comets, planets, and exoplanets.^6–8^ To convert this observational richness into direct information for the molecular physics of interest, requires models that can reliably translate line intensities into molecular abundances and temperatures. Crucial tools for such an interpretation are the collisional (de)-excitation rate coefficients for the dominant projectiles known to be abundant in those environments: chiefly He and H_2_. Such rate coefficients govern molecular level populations when the local thermodynamic equilibrium (LTE) approximation breaks down, e.g., in the low-density regimes typical of many astrophysical environments, e.g., see: ref 9. However, despite their fundamental role, reliable knowledge of collisional data remains scarce and often constitutes a major bottleneck for the interpretation of modern observations.

In the present work, we suggest the application of a computational reduction scheme which, unlike other methods (e.g., see: refs 3,4 ), does not simplify the channel coupling in the time-independent quantum scattering but reduces instead the dimensionality of the body-fixed coordinates by averaging over the orientations of the collision partner provided by the neutral H_2_ molecule in its *para* configuration. Because of the special nature of this partner molecule, we show that by averaging over its internal coordinates, we shall recover a two-dimensional (2D) representation of the PES that provides very realistic values for the relevant inelastic cross sections and rate coefficients. BN^–^, which is likely not found in the ISM, was chosen because of its relevance in laser-cooling applications^10–12^ which also initiated previous work on rotationally inelastic collisions of BN^–^ with H_2_ using a four-dimensional (4D) representation of the PES by some of us.^13^ In the next section, we will describe our proposed method, while our results are presented in the following section. Our present conclusions will be given in the last section.

## Methods

### Computed Interaction Forces

Quantum chemistry calculations were carried out via the *ab initio* method given in several references.^14–16^ Briefly, we employed the RHF-RCCSD­(T)/aug-cc-pV5Z choice of basis set, which was also corrected for the basis set superposition error (BSSE) along the selected grid of points.^17^ It included single, double, and triple noniterative excitation [CCSD­(T)].^18^

When considering the full representation in 4 dimensions (4D) of the Jacobi internal coordinates, the distance between the B atom and the center of mass (COM) of BN^–^ was taken to be 0.7272 Å, while 0.5612 Å was used as the N-COM distance. The bond distance in the neutral H_2_ molecule was kept fixed at 0.7400 Å. In our earlier work on the cooling of the C_2_^–^ anion in collisions with H_2_,^19^ we further considered the vibrating anion besides the rigid-rotor case, but this extension of the present calculations will be reported elsewhere.

Within the 4D choice of internal Jacobi coordinates, the distance between the two COMs is defined as *R* and there are three angular coordinates given as

(i) θ = the valence angle between the BN bond and the line connecting the COM­(H_2_) and the COM­(BN^–^) from the B side;
(ii) α = the valence angle between one atom of H_2_ and the line connecting the COM­(H_2_) and the COM­(BN^–^);
(iii) β = the torsional angle between H_2_ and the line connecting the COM­(H_2_) with the COM­(BN^–^) on the N side.

The case of θ = 0° corresponds to the orientation where the COM­(H_2_) is collinear with BN^–^ and on the side of the B atom, while the case with θ = 180° defines the COM­(H_2_) collinear with BN^–^ and on the side of the N atom. In both instances, the additional α angle is also = 0°. We have computed around 50 radial points for each selection of angular values for the (θ, α, β) angular set. The angle θ changes from 0° to 180° with 5° intervals. The angle α changes with variable intervals as explained below, while the angle β takes the values 0°, 45° or 90°, as also explained below. For θ = 0° or 180°, β is undefined, so we set it to 0. Hence, there are no data for β = 45° or 90°. For configurations, where H_2_ gets near to the N-end of the anion, we find that the structure is more stable. So, for θ = 175°, 150°, 120° and 90°, the angle α is incremented in 5° intervals while β is 0°, 45° and 90°. Such a selection is enough to span the whole space, since symmetry tells us that *V*(θ, α, 180°) = *V*(θ, 180° – α, 0°) and also that *V*(θ, α, 135°) = *V*(θ, 180° – α, 45°) and that *V*(θ, α, 90°) = *V*(θ, 180° – α, 90°). Additionally, for all other values of θ, α is increased by 15° and β = 90° plus a few cases where β = 45°. A total of 48,956 points were obtained when the full 4D calculations were carried out by us, as we reported in ref 13.

In Figure 1, we report on the relative orientation of the two molecules and the definition of the angles. The upper panel of Figure 1 shows the internal coordinates for the full 4D calculations of the body-fixed (BF) spatial representation of the PES used earlier.^13^ The lower panel, on the other hand, shows the reduced 2D Jacobi coordinates after averaging, as described below: it will be the Jacobi choice for the present dimensionality-reduction calculations.

**Figure 1:**
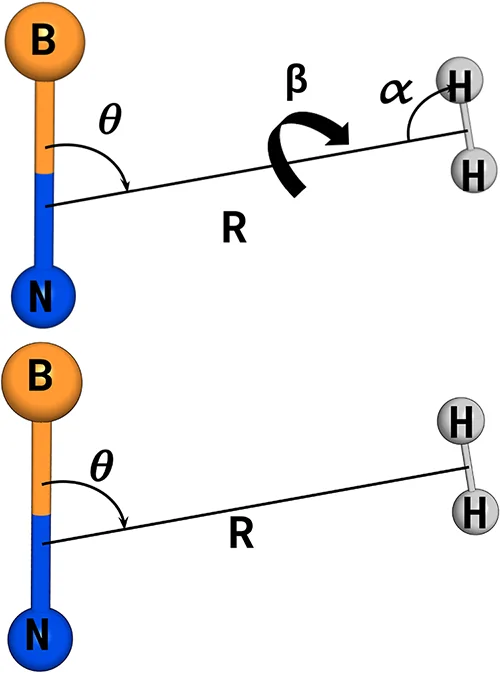
Pictorial view of the Jacobi coordinates for the BN^–^···H_2_ system. The 4D Jacobi coordinates (*R*, θ, α, β), are given in the upper panel while the lower panel shows the 2D coordinates (*R*, θ) left after the arithmetic average of the α and β angles, as defined in the main text.

The next step in treating the raw points of the *ab initio* calculations outlined earlier, was that of obtaining an artificial neural network (ANN) fit of those points, as described in detail in many of our earlier works,^13,19,20^ so we refer to those papers for further details. We performed the fit to obtain a PES invariant under permutation of like atoms. This was done using the fundamental invariant (FI) basis, as described in refs 13,20 , so that we will not repeat it here.

For the ANN procedure, the total data set with energies less than 5000 cm^–1^ (45,819 points) was split into a training/validation/test ratio of 70:15:15. The training data included randomly selected points up to an energy of 3000 cm^–1^ and all energy points in the 3000–5000 cm^–1^ range. The NeuralNetwork module of PES-Learn was then used to train the PES.^21^ A series of neural network architectures, double-hidden-layer and triple-hidden-layer models, with each network trained for up to 1000 epochs using the LBFGS optimizer, was tried. The best-performing network was identified based on its full-data root-mean-squared deviation (RMSD) (*V* ≤ 5000 cm^–1^), and this network was subsequently trained for a maximum of 10,000 epochs using different random-number seeds for weight initialization. The hyperparameters used in the best-performing model were as follows: the neural network architecture consisted of three hidden layers with 160, 80, and 20 neurons, respectively. The hyperbolic tangent (tanh) was used as activation function. Both the input features and target values were scaled using the standardization (std) method, and the learning rate was set to 0.5. The RMSD of the final potential on the full-data set was 1.25 cm^–1^, and for the energy range <3000 cm^–1^, the RMSD was found to be 1.09 cm^–1^.

### Theoretical Modeling

The basic choice of the present model is averaging over two of the 4D coordinates discussed in the previous section to produce a reduced-dimensionality (2D) representation of the BN^–^···H_2_ system. Thus, we generated a two-dimensional PES, *V*(*R*, θ), by averaging over the three orientations, as performed in our earlier work^19,20,22^

1 $$V_{\mathrm{av}}\left(R,\theta \right)=\frac{1}{3}\left[V_{\mathrm{perp}}\left(R,\theta \right)+V_{\mathrm{in}‐\mathrm{line}}\left(R,\theta \right)+V_{\mathrm{oop}}\left(R,\theta \right)\right]$$

The orientations considered were: (i) α = 90°, β = 0 (the “perp” orientation), (ii) α = 0, β = 0 (the “in-line” orientation), and (iii) α = 90°, β = 90° (the out-of-plane, “oop” orientation). The final view of the resulting, reduced-dimensionality potential coordinates is presented in the lower panel of Figure 1. This treatment of the interaction with the H_2_ partner is physically equivalent to restricting the latter molecule to be only *para*-H_2_ in its *j* = 0 level and has been already used in the past for various other systems, as we and others discussed in earlier work.^3,22^ In Figures 2 and 3, we present two different pictorial views of the general quality of the ANN fitting to the averaged 2D points and a further presentation of the spatial anisotropy of the reduced-dimensionality 2D PES in polar coordinates.

**Figure 2:**
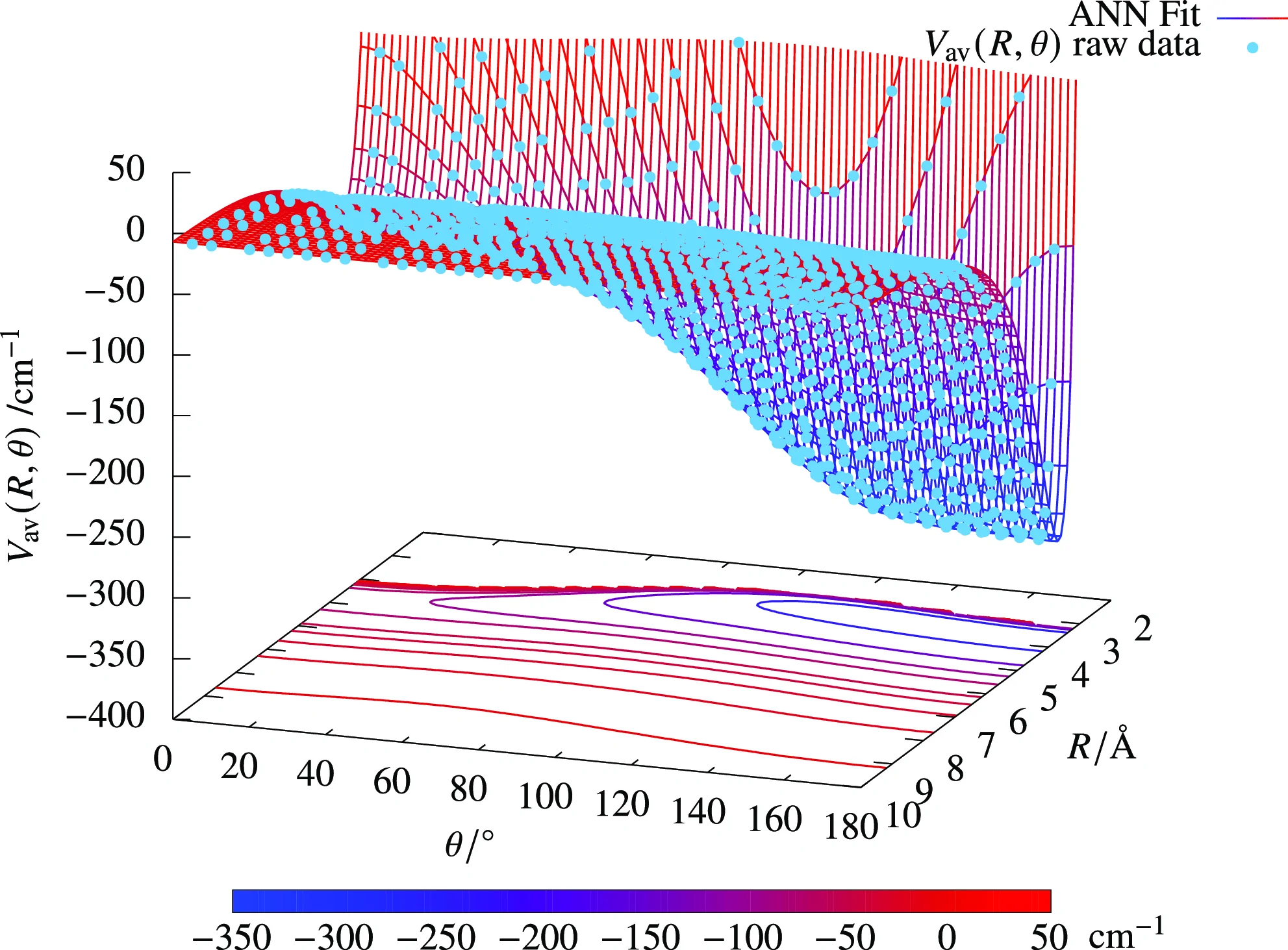
Plot of the potential energy behavior for the 2D (*R*, θ) variables describing the BN^–^···H_2_ system using the averaged raw data and the ANN fitting curves, respectively. Distances are given in units of Å and energies in cm^–1^.

**Figure 3:**
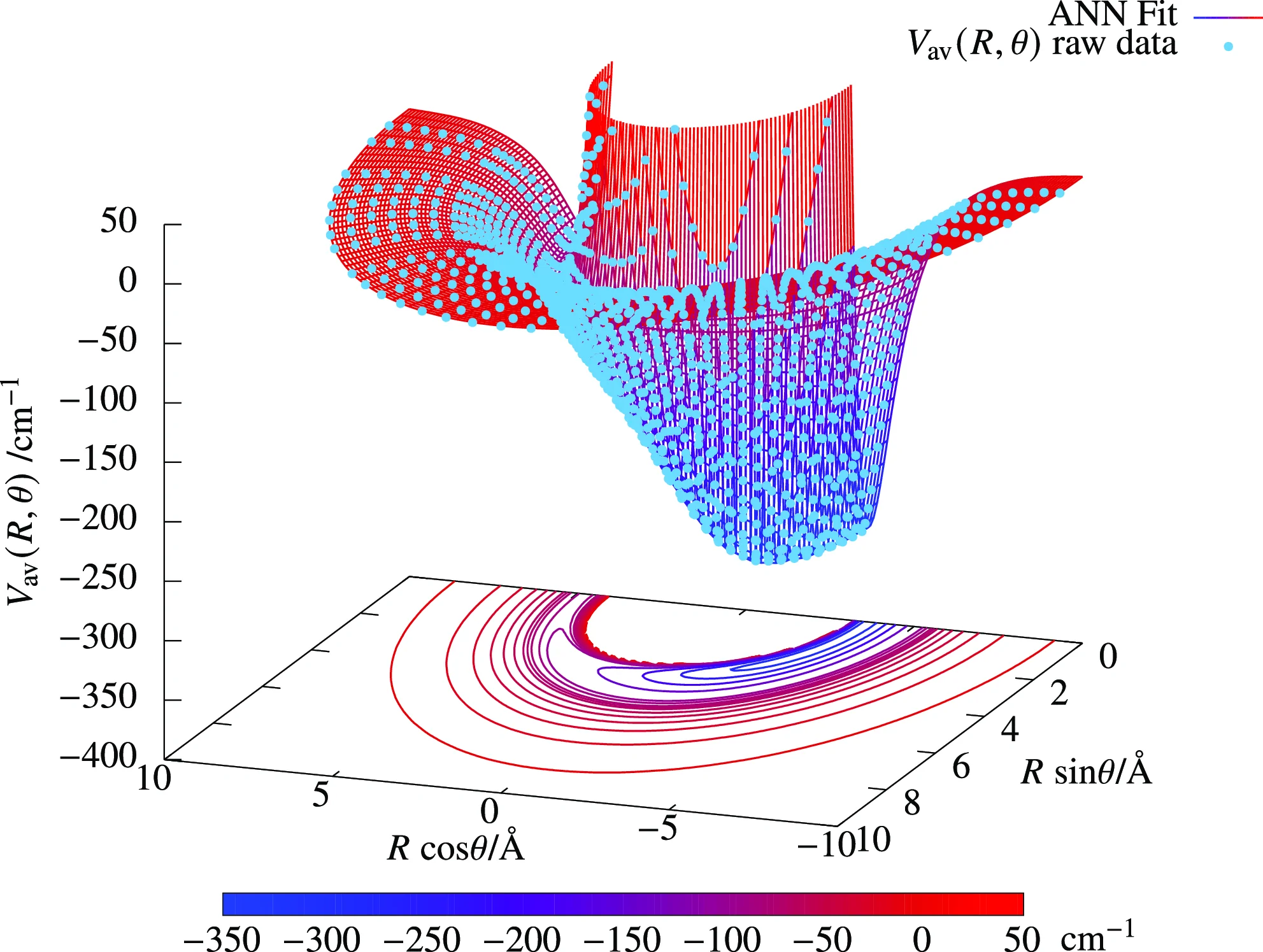
Plot of the potential energy of the BN^–^···H_2_ system as a function of the spatial orientation, i.e., for the 2D *R* sin θ and *R* cos θ variables, using the averaging discussed in eq 1. Distances are given in units of Å and energies in cm^–1^.

The long-range (LR) behavior of the interaction is

2 $$V_{\mathrm{LR}}=-\frac{\alpha_{0}}{2R^{4}}-\left(\frac{2\alpha_{0}\mu }{R^{5}}\right)\cos\theta$$

where α_0_ = (α_∥_ + 2α_⊥_)/3. The polarizability of H_2_ at *r* = 1.4 *a*_0_ (0.7408 Å)^23^ is given by α_0_ = 5.1786 *a*_0_^3^ and α_2_ = 1.8028 *a*_0_^3^. Since the first term dominates the interaction, we shall keep only the spherical polarizability form in our LR potential term.

The interaction potential in this reduced 2D form was expanded in orthogonal Legendre polynomials for the angle θ, so that it can be described via two variables only

3 $$V\left(R,\theta \right)=\sum V_{\lambda }\left(R\right)P_{\lambda }\left(\cos\theta \right)$$

A pictorial view of the multipolar coefficients for the ensuing 2D representation of eq 1, which now depend only on the (*R*, θ) variables, is shown by the radial terms in Figure 4. One clearly sees in Figure 4 that, next to the strongest radial coefficient for the spherical term with λ = 0, the anisotropic, direct coupling comes from the λ = 2 contribution, a feature which will be reflected on the relative sizes of the inelastic cross sections later in this work.

**Figure 4:**
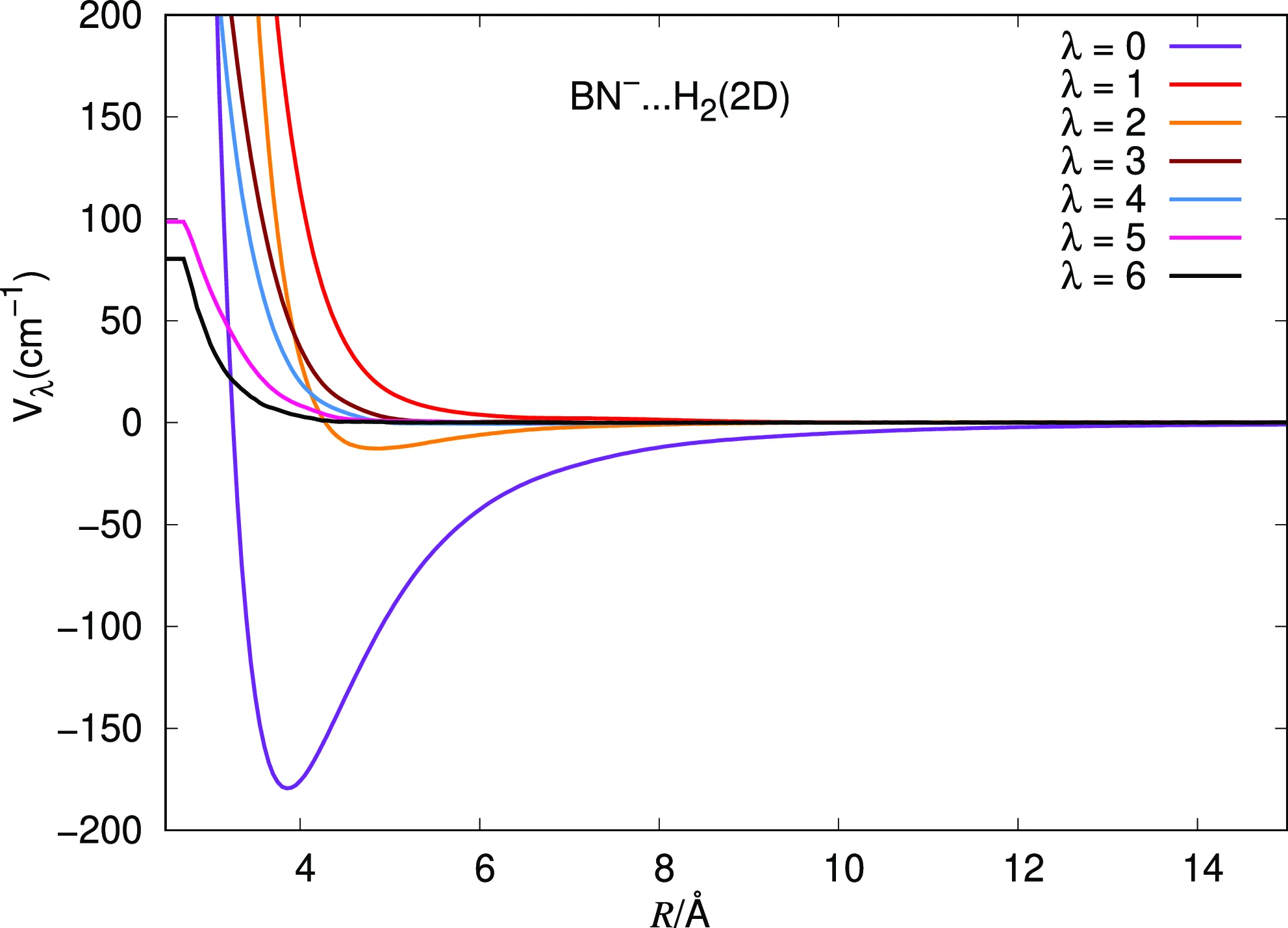
Plot of the computed multipolar coefficients defined in eq 3 obtained using the averaging discussed in eq 1. The plot shows their behavior for the 2D (*R*, θ) variables now describing the BN^–^···H_2_ system. Distances are given in units of Å and energies in cm^–1^.

Since the formulation of the ensuing CC equations has been given many times before (e.g., see: refs 24,25 ), we shall not repeat it here, and provide only the final expression for the inelastic, state-to-state cross section for the case in which the full CC equations are solved within the 2D formulation of the scattering problem, as discussed in our earlier work^12^

4 $$\left(\frac{d^{2}}{dR^{2}}+K^{2}-V-\frac{l^{2}}{R^{2}}\right)f^{J}=0$$

Here, each element of **K** = δ_*i*,*j*_2 μ (*E* – ϵ_*i*_) (where ϵ_*i*_ is the channel asymptotic energy and μ is the reduced mass of the system), **V** = 2 μ**U** is the interaction-potential matrix between channels and **l** ^2^ is the matrix of orbital angular momentum. In line with many earlier calculations by us,^12,19,26^ we have treated the doublet-state of the target anion as a pseudosinglet, an efficient simplification that has been found to also yield little difference in comparison with the correct inclusion of spin–orbit coupling.^22,27^ Scattering observables are obtained in the asymptotic region so that, at the end of the propagation, one can use the log-derivative matrix to obtain the **K**-matrix by solving the following linear system

5 $$\left(N^{\prime }-\mathrm{YN}\right)=J^{\prime }-\mathrm{YJ}$$

where **J**(*R*) and **N**(*R*) are matrices of Riccati-Bessel and Riccati-Neumann functions.^28^ The **S**-matrix is easily obtained from the **K**-matrix, and the state-to-state cross sections are then calculated from the Scattering **S**-matrix as

6 $$\sigma_{j\to j\prime }=\frac{\pi }{\left(2j+1\right)k_{j}^{2}}\sum_{J}\left(2J+1\right)\sum_{l,l^{\prime }}{\left|\delta_{\mathrm{lj},l\prime j\prime }-S_{\mathrm{lj},l\prime j\prime \prime }^{J}\right|}^{2}$$

To converge the CC equations, the rotational basis set included states up to *j* = 29 within the CC expansion. The CC equations were propagated between 1.0 and 15.0 Å using the log-derivative propagator^29,30^ with a step size of 0.08 Å and, at higher values up to 170.0 Å, using the variable-phase Airy propagator^25^ for a total of 1000 steps. The convergence of the scattering calculations with respect to the basis set, number of terms in the PES expansion, propagation distance and number of steps was checked. The cross sections are converged to at least a few percent or better, which will have a negligible effect on calculated rates. Hence, the rotational-state-changing cross sections are directly obtained for the rotating anion, as indicated in eq 6. We note once more that, with regard to the spin–orbit-coupling terms, the above scattering formulation treats the BN^–^ anion as a pseudosinglet collision partner, a simplification which we have already discussed earlier and tested in several works of our group (e.g., see: ref 22).

The calculations were carried out following the above prescription as implemented in MOLSCAT, a well-known software for quantum scattering calculations,^29,30^ and the results will be reported in the following section. We also computed the inelastic rate coefficients. These were obtained via the usual quadrature formula over the relevant cross sections which were defined above

7 $$k_{j\to j\prime }\left(T\right)={\left(\frac{8}{\pi \mu k_{B}^{3}T^{3}}\right)}^{1/2}\times \int_{0}^{\infty }\sigma_{j\to j\prime }\left(E_{\mathrm{coll}}\right)E_{\mathrm{coll}}e^{-E_{\mathrm{coll}}/k_{B}T}dE_{\mathrm{coll}}$$

where the meaning of the various quantities has been given before. Only the rotational-state-changing processes for the anion have been considered. The numerical integration was checked for the convergence of the final rate-coefficient values at different points of the required range of temperatures presented in the following section.

## Results and Discussion

Figure 5 shows the results for the inelastic cross sections involving excitation and de-excitation transitions between the lowest five levels of the BN^–^ anion. The present results, given by the black lines, are compared with the results of our earlier calculations using the full 4D dimensionality^13^ for the *para*-H_2_ partner (red lines). The upper panel of that figure shows excitation processes, while the lower panel presents de-excitation processes. It is evident from the comparison that the two sets of cross sections turn out to be essentially the same in size and largely show the same energy dependence. Hence, the exact, CC calculations with the 4D full interaction are coinciding with the dimensionality reduction approach of the black curves, also obtained via a full CC scattering calculation.

**Figure 5:**
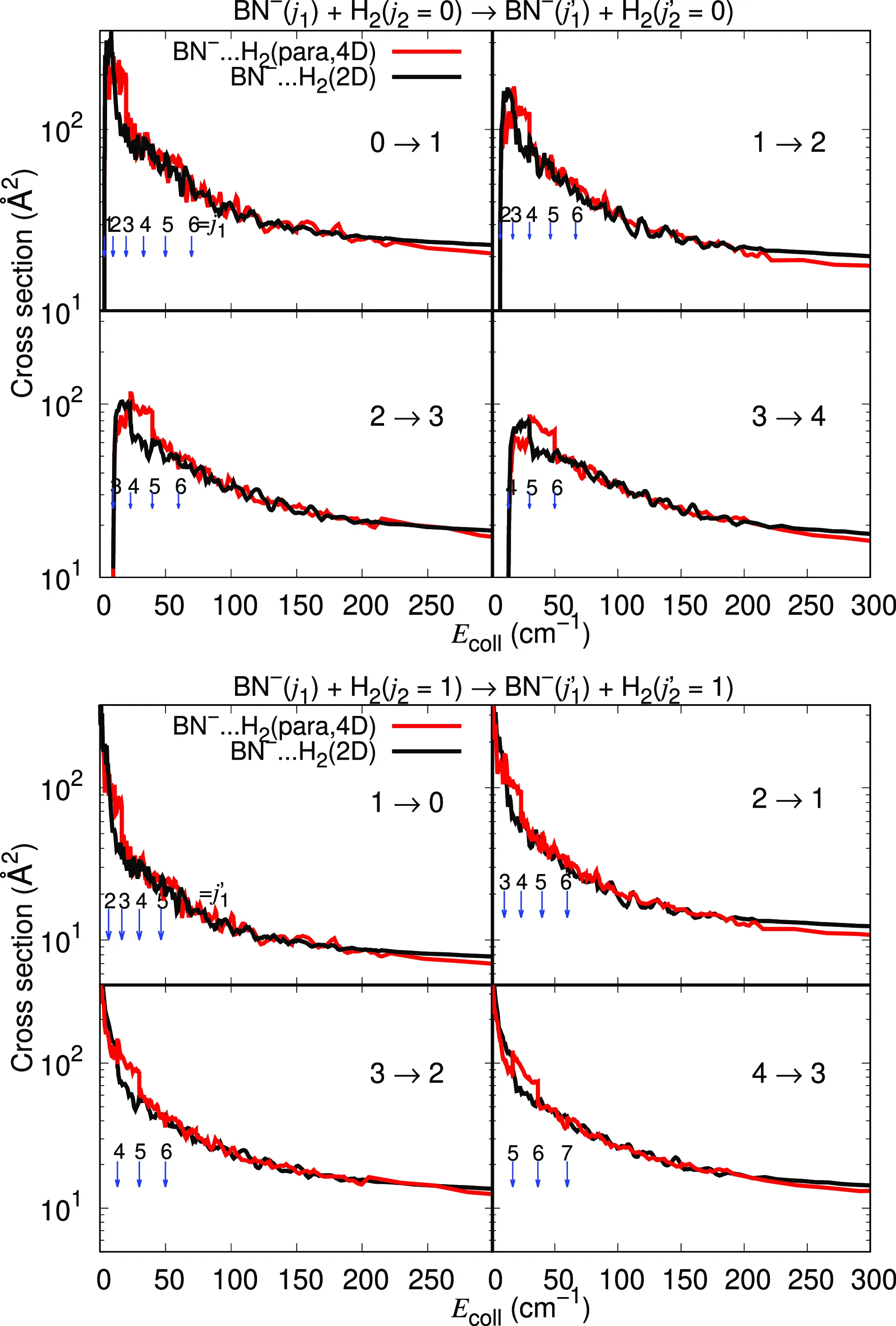
Upper panel: Excitation cross sections plotted as a function of collision energy *E*_coll_ for a variety of initial rotational levels of BN^–^ in collision with H_2_. The red lines show results from ref 13 where the exact 4D calculations were done. The black lines show the present results obtained using the 2D dimensionality reduction. Lower panel: The same set of processes but considering the de-excitation cross sections.The arrows in each panel mark the opening of the upper channels as the energy increases. See main text for further details.

More comparisons are reported in the panels of Figure 6, where the rotational states between transitions are now rotationally excited levels of the anion. The transitions between excited states naturally imply larger energy gaps between the levels and, therefore, the corresponding inelastic cross sections, both for the excitation and de-excitation state-changing channels, are now uniformly smaller, as is to be expected. Furthermore, we have a clear confirmation that, even for these transitions, the 2D and 4D choices of interaction forces show very little changes and indeed turn out to be nearly coincident. These results indicate that the averaging procedure involving the orientations of the neutral partner H_2_ has a limited effect on the efficiency of the state-changing collisions. Here, the physics of the rotational coupling is chiefly controlled by the orientational anisotropy of the main interaction, given by the (θ, *R*) Jacobi variables of the PES.

**Figure 6:**
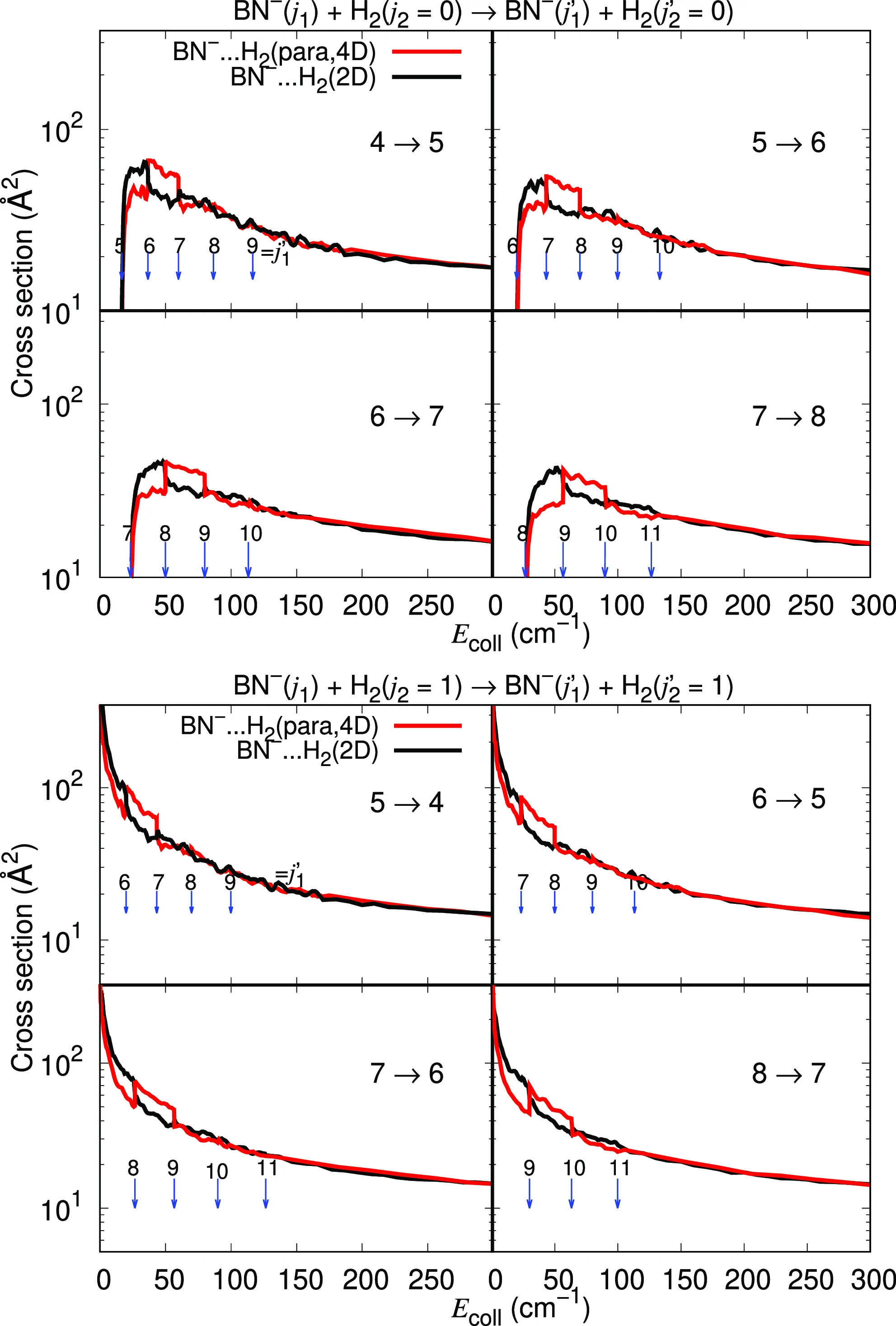
Upper panel: Excitation cross sections plotted as a function of collision energy *E*_coll_ for higher excited rotational levels of BN^–^. The red lines show results from ref 13 in which exact 4D calculations were carried out. The black lines show the present results obtained using the 2D dimensionality reduction. Lower panel: The same set of processes but considering the de-excitation cross sections instead. The arrows mark the openings of higher channels as the energy increases. See main text for further details.

One should note that in the lower energy regimes both sets of cross sections exhibit a large number of oscillations which get washed out when the inelastic rate coefficients are computed (see below). Such features are due to the occurrence of resonant effects either from open channel or closed channel coupling during collisions (for a more extensive discussion, see: refs 24,25 ). They are also due to threshold resonances due to the opening of upper channels becoming open as the energy increases.One sees that such oscillations decrease as features as one moves in energy to higher channels further away from the one involved in the primary transitions: the coupling strength gets in fact reduced as the channel considered is further away from that of the primary process.

The reduced-dimensionality calculations lead to substantial savings in computational effort. The data reported in Table 1 provide quantitative estimates of such savings. These data show that the 2D calculations provide, on average, a 2 orders of magnitude reduction of computer times compared to the 4D CC calculations, this being generally true across a broad range of collision energies and a variety of state-changing inelastic channels. It therefore follows that, given this invariance of the inelastic cross section values, the 4D → 2D dimensionality reduction in the case of the H_2_ collision partner essentially leaves the quality of the collision physics unchanged. As for the hardware involved, both sets of calculations were run on the same machines. Briefly: on a x86_64 architecture, with an address size of 46 physical bits and 40 CPUs on line, with 64 GB RAM. More specifically, the model was an Intel­(R)­Xeon­(R)-CPU E5–2650v3@2.30 GHz, with 2 threads per core and 10 cores per socket. The CPU maximum frequency was 30 × 10^6^Hz.

**Table 1:** Computing Time Analysis for the Cross Section Values for BN^–^ + H_2_ (2D and 4D Calculations) Using MOLSCAT^30^

		Total time taken (CPU seconds)		Average time taken for a single inelastic channel (CPU seconds)	
*E*_coll_ (cm^–1^)	Total number of inelastic channels	2D	4D	2D	4D
3.4	2	13.71	3447.38	**6.86**	**1723.69**
10.0	6	194.90	14,770.06	**32.48**	**2461.68**
20.0	12	409.32	24,692.41	**34.11**	**2057.70**
35.0	20	590.28	32,845.43	**29.51**	**1642.27**
50.0	30	805.25	48,449.14	**26.84**	**1614.97**
70.0	42	1021.74	63,763.30	**24.33**	**1518.17**
100.0	56	1266.92	73,212.13	**22.62**	**1307.36**
150.0	90	1851.94	135,210.98	**20.58**	**1502.34**
200.0	110	3029.67	163,990.43	**27.54**	**1490.82**
300.0	156	5622.87	280,233.30	**36.04**	**1796.37**
500.0	650	12,763.68	666,514.89	**19.64**	**1025.41**

To report more quantitative indicators for the similarity which exist between the 4D and 2D sets of computed cross sections, we plot in the panels of Figure 7 the values of their residuals involving one of the groups of transitions we have considered in this study. The values for the case of the more excited state transitions are given in the files of the Supporting Information. One clearly sees that both sets of cross sections are practically coincident (residuals close to zero) as the energy gets close to 50 cm^–1^ and all the way up to the highest energies considered. On the other hand, the larger scattered values occur near threshold for both the excitation and de-excitation processes. Furthermore, looking at the total RMSD values, also reported by each panel, we see that they all stay below 30 Å^2^. Considering that at such energies the actual cross sections are larger than 10^2^,this indicates the high level of value similarity with the correct calculations which comes from the present 2D approximation within exact CC quantum dynamics. In term of percentage values, therefore, the differences between 2D and 4D calculations remain well below 20% at all energies. This means that the final rate coefficients, as shown below, remain all within the same order of magnitude: a crucial requirement for using them within kinetic modeling processes.

**Figure 7:**
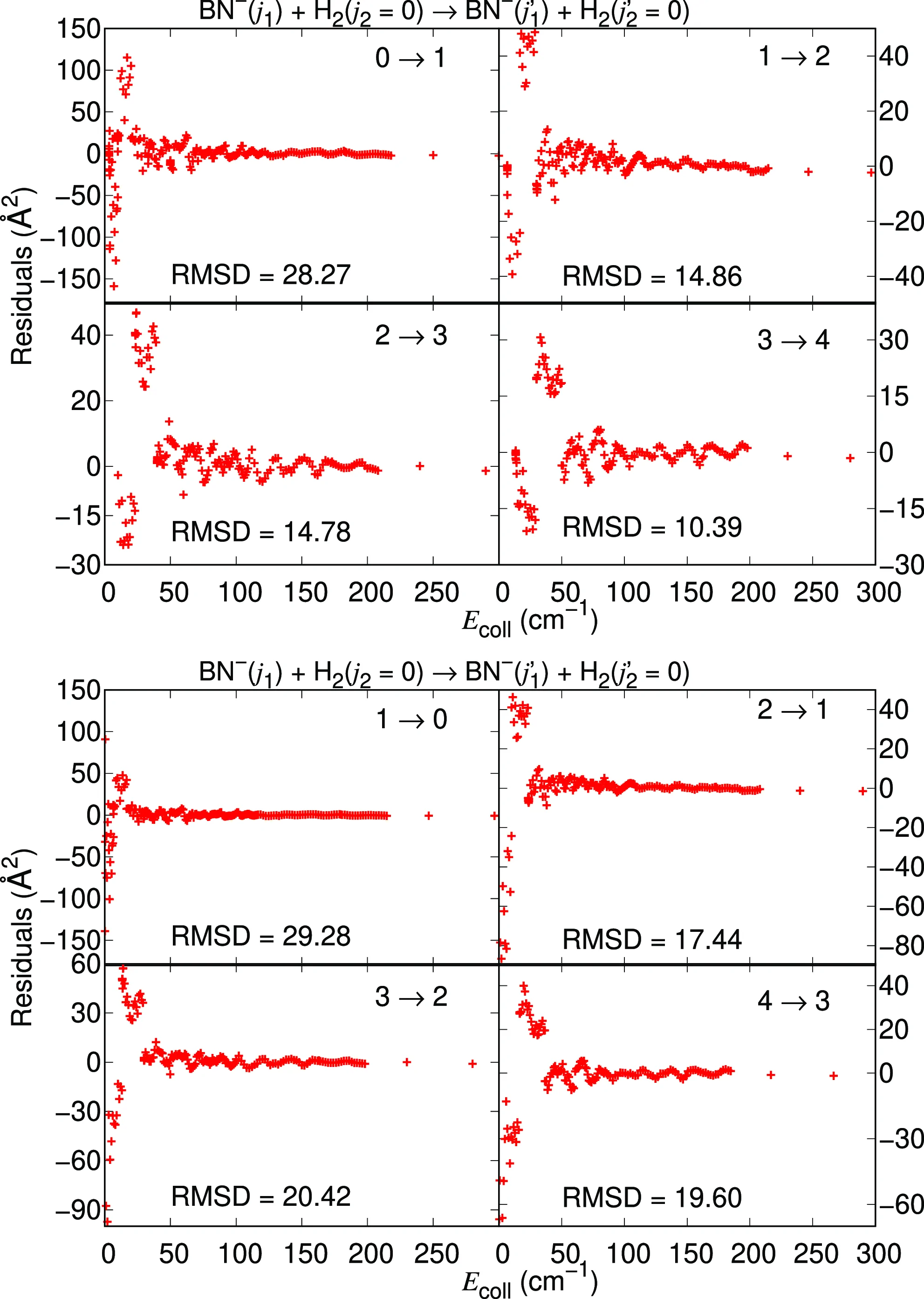
Upper panel: Excitation cross sections as a function of collision energy *E*_coll_ for the lower rotational levels of BN^–^. Lower panel: The same set of processes but considering the de-excitation cross sections. The red crosses report the values of the residuals, and also show the Root-Mean-Square Deviation (RMSD), between the sets of transitions. See main text for further details.

The panels in Figures 8 and 9 show the results of our calculations for the inelastic rate coefficients. In all the panels in the two figures, we see a clear confirmation of the fact that the dimensionality reduction via the averaging reported in the previous section does not change the rotational-state-changing probabilities for the BN^–^ anion colliding with *para*-H_2_. The latter has been chosen not only because of its use as a different buffer gas in cold ion traps, but also because it is the most abundant partner in molecular clouds with the variety of anions and cations detected there. Hence, to be able to save computational time for generating dynamics attributes of interest in LTE analysis (e.g., see ref 31), which are shown here to be unchanged from the cases where the 4D interaction is employed, becomes an important asset in astrochemical modeling.

**Figure 8:**
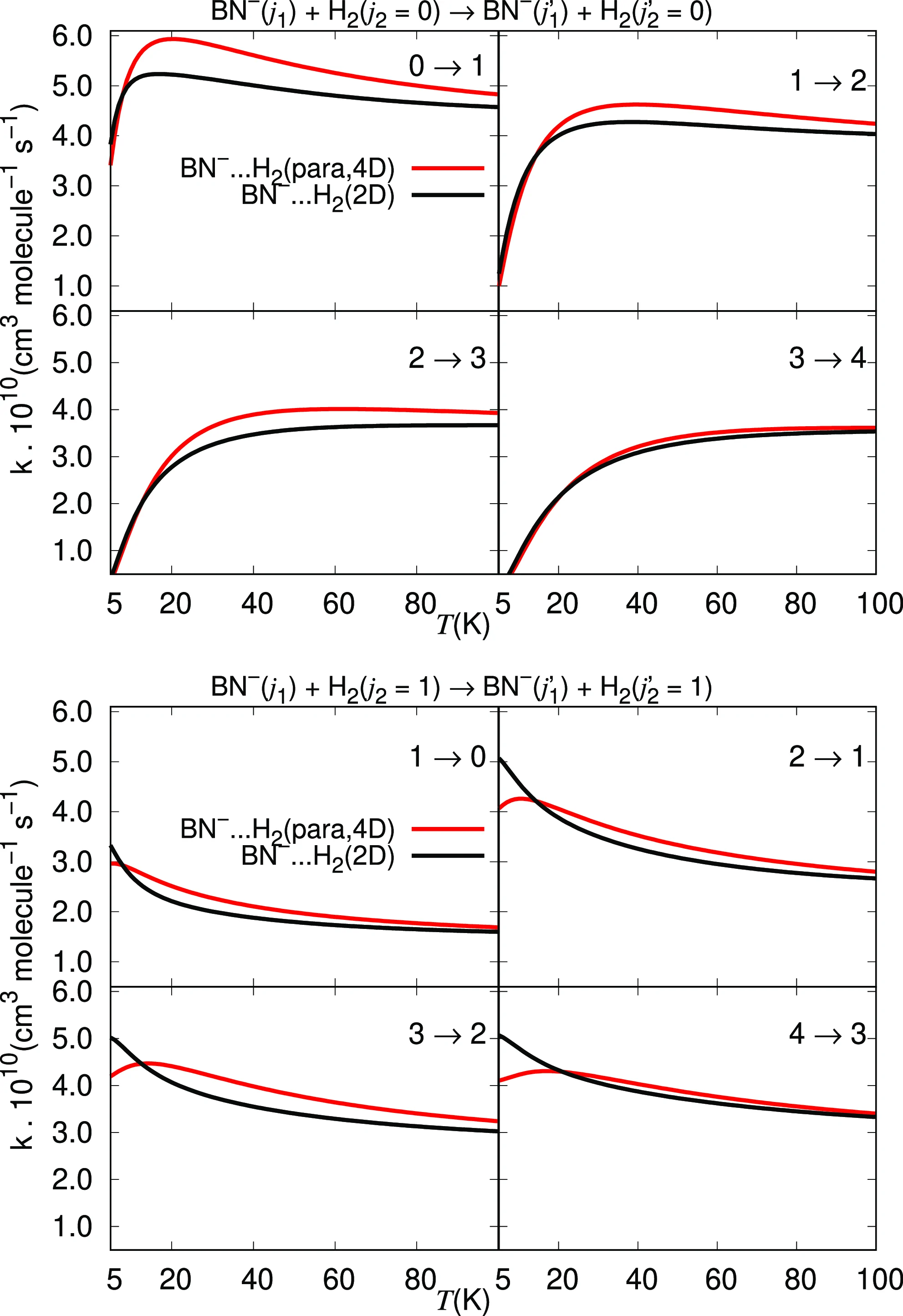
Upper panel: Excitation rate coefficients as a function of temperature *T* for a variety of initial rotational levels of BN^–^ in collision with a buffer gas. The red lines show results from ref 13 in which H_2_ is treated as *para*-H_2_. The black lines show the present results obtained using the 2D dimensionality reduction. The Δ*j* = 1 selection rule is considered for the excitation processes. Lower panel: The same set of processes but considering the de-excitation rate coefficients instead. See main text for further details.

**Figure 9:**
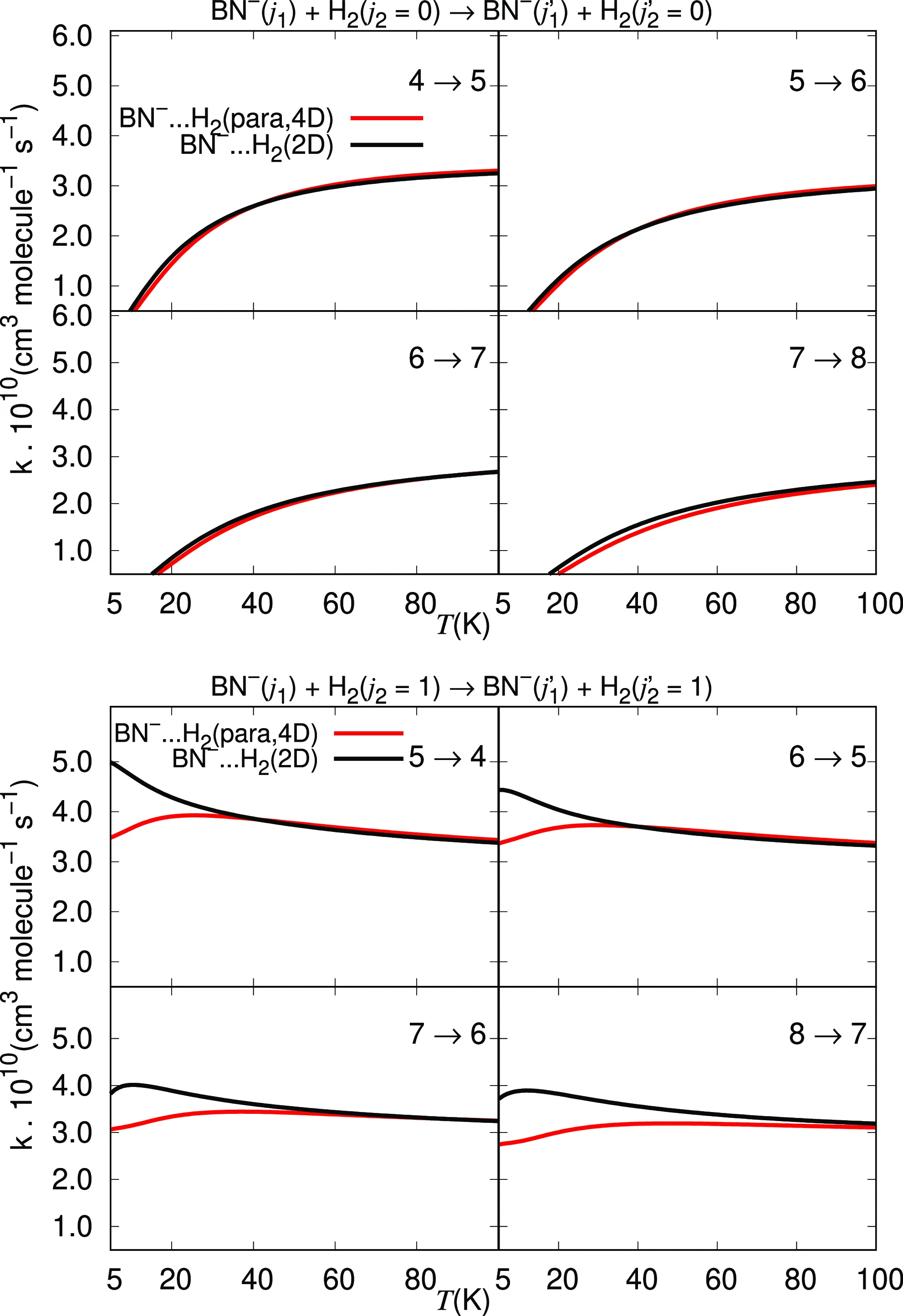
Upper panel: Excitation rate coefficients as a function of temperature *T* for a variety of excited rotational levels of BN^–^ in collision with a buffer gas. The red lines show results from ref 13 in which H_2_ is treated as *para*-H_2_. The black lines show the present results obtained using the 2D dimensionality reduction. The Δ*j* = 1 selection rule is considered for the excitation processes. Lower panel: The same set of processes but considering the de-excitation rate coefficients instead. See main text for further details.

By further looking at the panels of Figure 9, which involve transitions between excited states of the anion, we are able to confirm what was shown in the earlier figures. We also see that the differences between the two sets of results become smaller for the transitions with the largest energy gaps and at the lowest temperatures. On the other hand, the largest differences are still within about 20%, which is an acceptable difference for modeling studies.^32^ We also wish to point out that, in our recent study of rotationally inelastic collisions in the C_2_^–^···H_2_ system,^19^ a detailed comparison between computed inelastic cross sections and rates also showed unequivocally that the dimensionality reduction of the PES yielded results essentially coincident with those using the full 4D PES.

## Conclusions

In the present work, we have endeavored to show that, when dealing with rotationally inelastic collisions between a molecular anion and the neutral molecule H_2_ in its ground rotational state, it is possible to achieve a substantial reduction of the computational times without significant loss of accuracy for the final dynamical observables. We have shown this using the BN^–^ anion as an example. By averaging the accurate 4D PES over the orientational angles of the H_2_ partner, and therefore by treating the collision partner of the anion as a pseudospherical *para*-H_2_ molecule, we find final results to be essentially unchanged. Since the direct structural source of anisotropic interaction, described by the remaining (θ, *R*) Jacobi coordinates, remains unchanged, it follows that the dynamical torque applied during low-temperature collisions, those of interest in both cold ion traps and astrophysical environments, remains realistically described during the quantum scattering computations.

Our detailed calculations for the system reported in this work, and similar tests conducted earlier by us and others in other systems,^3,19,20^ indeed confirm that the specific structural features of the H_2_ collision partner describe correctly the collisional anisotropy causing rotational-state-changing state-to-state probabilities even when a portion of the Jacobi coordinates involving this molecule are orientation-averaged, as discussed in the previous section.

It is still not yet clear to us whether such a procedure can be generalized to any type of diatomic or linear collision partner of the anionic molecules of interest, for instance, in the ISM environment. In fact, more computational tests would be needed for other possible astrochemical systems like CO, CO^+^ and CO_2_, all known to be fairly abundant in the interstellar medium albeit not as abundant as the H_2_ partner. Our present results confirm that the 4D → 2D reduction of the PES works very well for that molecule, which is in any case the most abundant molecular partner in ISM environments and therefore most likely to provide collision-driven processes of significant values for kinetic modeling studies. From the physical standpoint, in fact, the H_2_ projectile corresponds to a highly polarizable partner for which, in the case of collisions with ionic molecules, the spherical polarizability of eq 2 dominates the LR interaction. Hence, the spatial approaches to the anion, controlled by the variations of the (θ, *R*) coordinates, are still realistically guiding rotational inelasticity after the previous (α, β) averaging from eq 1.

## Supplementary Material



## Notes

The Fortran routine generating the NN PES in 2D; a file with the computed cross sections; a file with the computed inelastic rate coefficients; computed residuals and RMSD values; percentage differences plots of the inelastic cross sections (4D to 2D) (ZIP)
