# Supplementary material for: Testing a Potential Averaging for Quantum Inelastic Dynamics
Source: J Phys Chem A. 2026 Sep 23;130(40):8036–43. doi: 10.1021/acs.jpca.6c03968 (PMC13656785; doi:10.1021/acs.jpca.6c03968)
Supplement: Supplementary file 1 [file jp6c03968_si_001.zip › S.I. BNm:H2 JPCA/%deviations_Xsecs/4d2d_bnmh2_exc_xsec_perdiff_2.pdf]

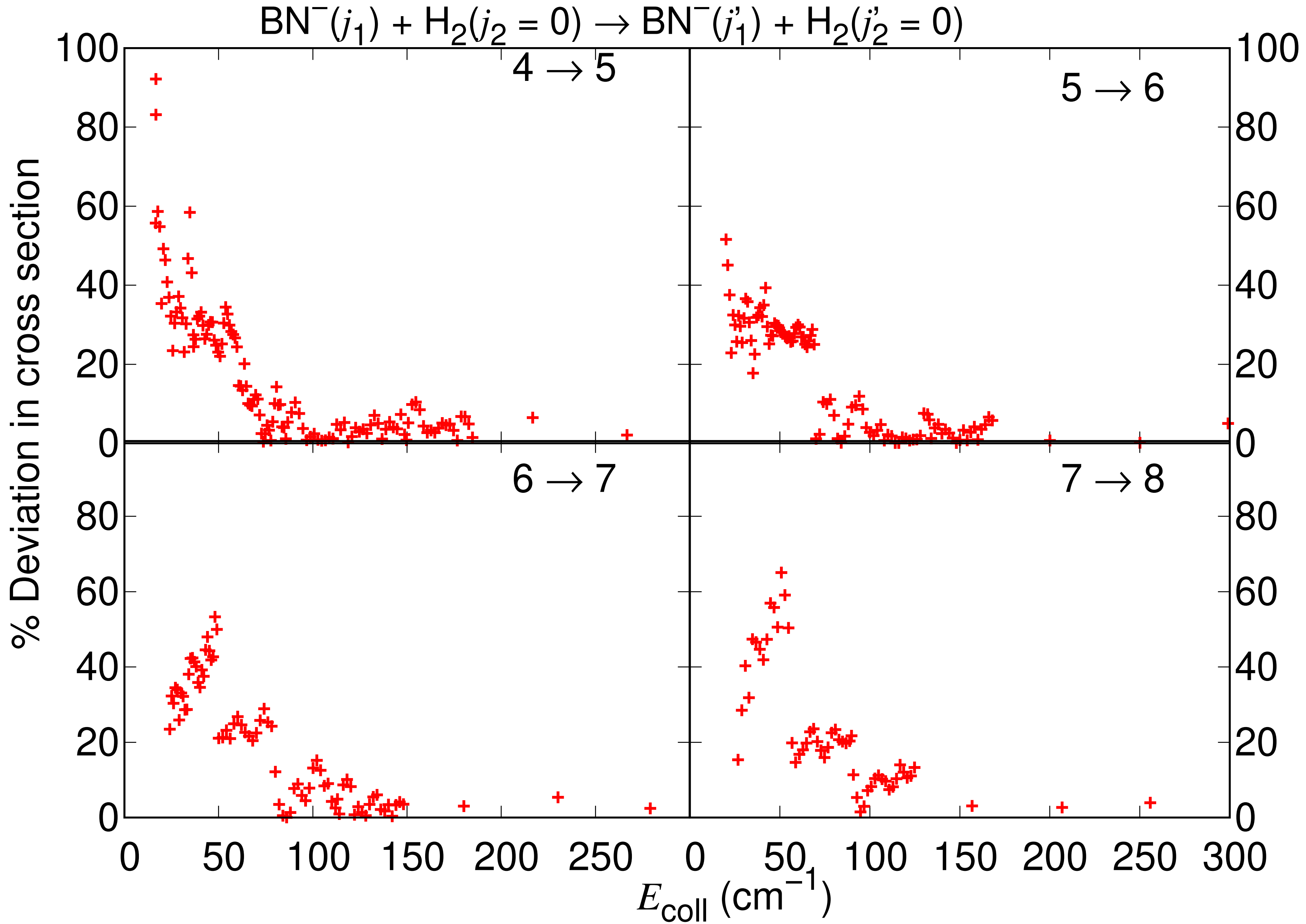

BN⁻(j₁) + H₂(j₂ = 0) → BN⁻(j'₁) + H₂(j'₂ = 0)
4 → 5
5 → 6
6 → 7
7 → 8
% Deviation in cross section
E_coll (cm⁻¹)
0
20
40
60
80
100
0
50
100
150
200
250
300
