# Supplementary material for: Testing a Potential Averaging for Quantum Inelastic Dynamics
Source: J Phys Chem A. 2026 Sep 23;130(40):8036–43. doi: 10.1021/acs.jpca.6c03968 (PMC13656785; doi:10.1021/acs.jpca.6c03968)
Supplement: Supplementary file 1 [file jp6c03968_si_001.zip › S.I. BNm:H2 JPCA/%deviations_Xsecs/4d2d_bnmh2_dexc_xsec_perdiff.pdf]

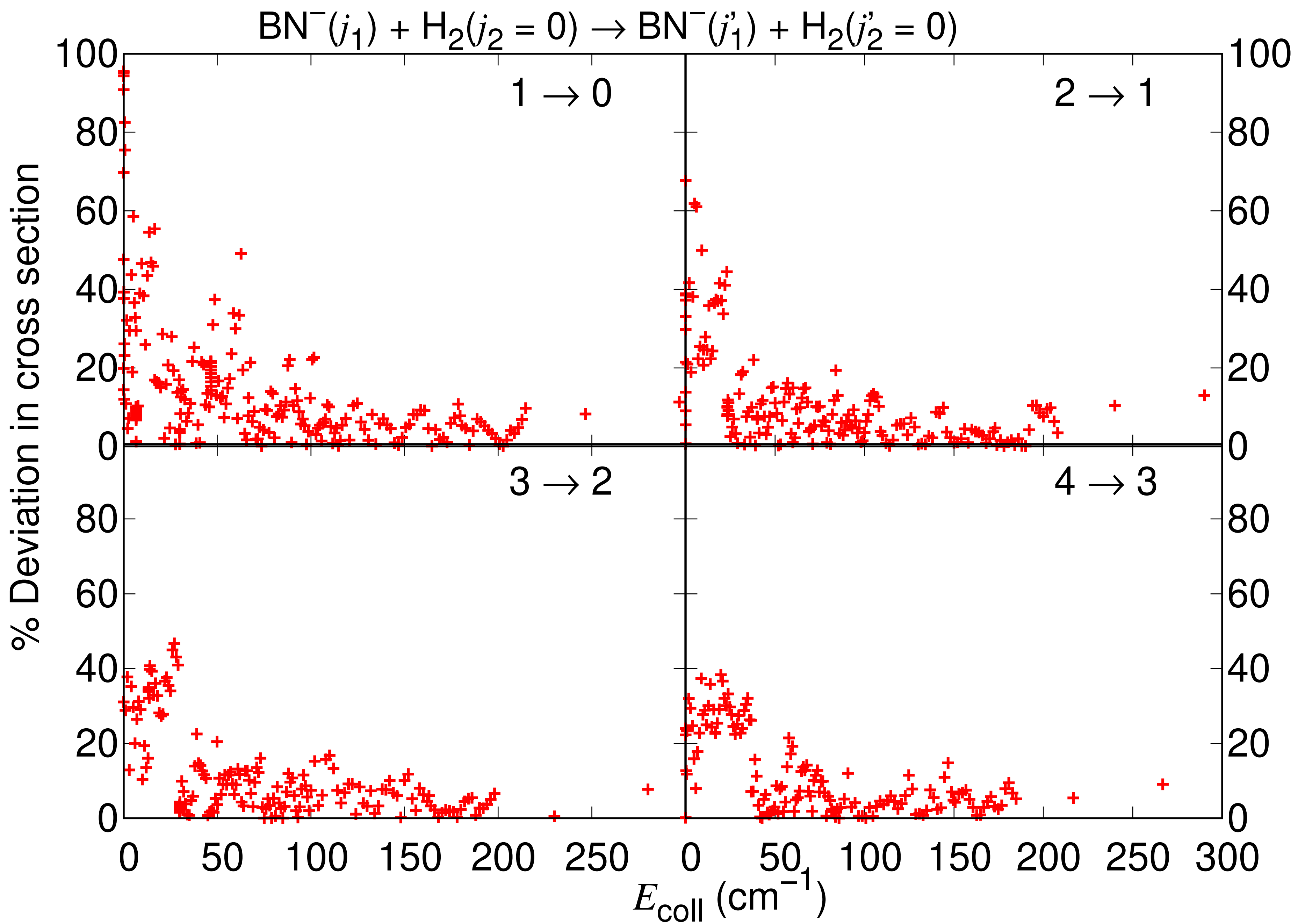

$BN^-(j_1) + H_2(j_2 = 0) \rightarrow BN^-(j'_1) + H_2(j'_2 = 0)$
$1 \rightarrow 0$
$2 \rightarrow 1$
$3 \rightarrow 2$
$4 \rightarrow 3$
% Deviation in cross section
E_{coll} (cm^{-1})
0 20 40 60 80 100
0 50 100 150 200 250 300
