# Supplementary material for: Testing a Potential Averaging for Quantum Inelastic Dynamics
Source: J Phys Chem A. 2026 Sep 23;130(40):8036–43. doi: 10.1021/acs.jpca.6c03968 (PMC13656785; doi:10.1021/acs.jpca.6c03968)
Supplement: Supplementary file 1 [file jp6c03968_si_001.zip › S.I. BNm:H2 JPCA/%deviations_Xsecs/4d2d_bnmh2_exc_xsec_perdiff.pdf]

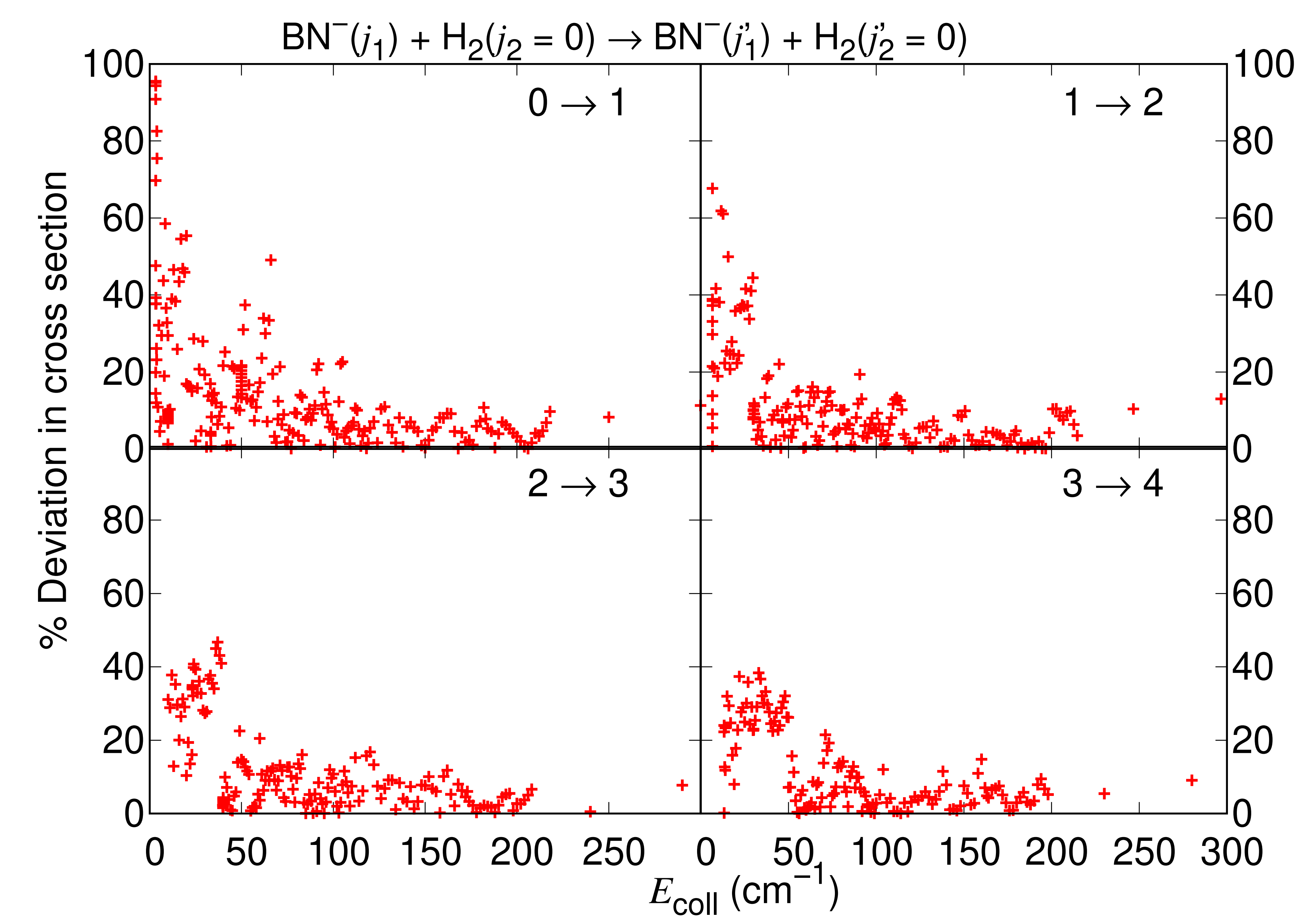

$BN^-(j_1) + H_2(j_2 = 0) \rightarrow BN^-(j'_1) + H_2(j'_2 = 0)$
0 → 1
1 → 2
2 → 3
3 → 4
% Deviation in cross section
E_{coll} (cm^{-1})
0
20
40
60
80
100
50
150
200
250
300
